# Myocardial strain assessment using cardiovascular magnetic resonance imaging in recipients of implantable cardioverter defibrillators

**DOI:** 10.1186/s12968-021-00806-4

**Published:** 2021-10-21

**Authors:** Nigel S. Tan, Djeven P. Deva, Kim A. Connelly, Paul Angaran, Iqwal Mangat, Laura Jimenez-Juan, Ming-Yen Ng, Kamran Ahmad, Vamshi K. Kotha, Joao A. C. Lima, Andrew M. Crean, Paul Dorian, Andrew T. Yan

**Affiliations:** 1 Division of Cardiology, Terrence Donnelly Heart Centre, St Michael’s Hospital, University of Toronto, 30 Bond Street, Toronto, ON M5B 1W8 Canada; 2 Department of Medical Imaging, St. Michael’s Hospital and Keenan Research Centre, Li Ka Shing Knowledge Institute, University of Toronto, Toronto, Canada; 3 Department of Medical Imaging, Sunnybrook Health Sciences Centre, University of Toronto, Toronto, Canada; 4 Department of Diagnostic Radiology, The University of Hong Kong, Hong Kong, China; 5 University of Calgary, Calgary, Canada; 6 Division of Cardiology, Johns Hopkins University School of Medicine, Baltimore, MD USA; 7 The Russell H. Morgan Department of Radiology and Radiological Sciences, Johns Hopkins University School of Medicine, Baltimore, MD USA; 8 Department of Epidemiology, Johns Hopkins University School of Public Health, Baltimore, MD USA; 9 University of Ottawa Heart Institute, Ottawa, Canada

**Keywords:** Cardiovascular magnetic resonance imaging, Left ventricular strain, Implantable cardioverter defibrillator, Outcome

## Abstract

**Background:**

Cardiovascular magnetic resonance (CMR) is increasingly used in the evaluation of patients who are potential candidates for implantable cardioverter-defibrillator (ICD) therapy to assess left ventricular (LV) ejection fraction (LVEF), myocardial fibrosis, and etiology of cardiomyopathy. It is unclear whether CMR-derived strain measurements are predictive of appropriate shocks and death among patients who receive an ICD. We evaluated the prognostic value of LV strain parameters on feature-tracking (FT) CMR in patients who underwent subsequent ICD implant for primary or secondary prevention of sudden cardiac death.

**Methods:**

Consecutive patients from 2 Canadian tertiary care hospitals who underwent ICD implant and had a pre-implant CMR scan were included. Using FT-CMR, a single, blinded, reader measured LV global longitudinal (GLS), circumferential (GCS), and radial (GRS) strain. Cox proportional hazards regression was performed to assess the associations between strain measurements and the primary composite endpoint of all-cause death or appropriate ICD shock that was independently ascertained.

**Results:**

Of 364 patients (mean 61 years, mean LVEF 32%), 64(17.6%) died and 118(32.4%) reached the primary endpoint over a median follow-up of 62 months. Univariate analyses showed significant associations between GLS, GCS, and GRS and appropriate ICD shocks or death (all p < 0.01). In multivariable Cox models incorporating LVEF, GLS remained an independent predictor of both the primary endpoint (HR 1.05 per 1% higher GLS, 95% CI 1.01–1.09, p = 0.010) and death alone (HR 1.06 per 1% higher GLS, 95% CI 1.02–1.11, p = 0.003). There was no significant interaction between GLS and indication for ICD implant, presence of ischemic heart disease or late gadolinium enhancement (all p > 0.30).

**Conclusions:**

GLS by FT-CMR is an independent predictor of appropriate shocks or mortality in ICD patients, beyond conventional prognosticators including LVEF. Further study is needed to elucidate the role of LV strain analysis to refine risk stratification in routine assessment of ICD treatment benefit.

## Background

The implantable cardioverter-defibrillator (ICD) reduces mortality in patients at high risk of sudden cardiac death. Randomized clinical trials and observational studies have established a role for ICD in patients with left ventricular (LV) systolic dysfunction and heart failure [1, 2], the prevention of recurrent cardiac arrest [3–5], and inherited conditions that predispose to sudden death [6]. However, many patients who meet the guideline-recommended criteria for ICD implantation never receive appropriate therapy for ventricular arrhythmias [7], and further risk stratification would be helpful.

Myocardial strain imaging has emerged as a useful clinical tool that can provide incremental information about LV systolic function beyond global assessment by LV ejection fraction (LVEF). LV strain imaging is a validated method of assessing regional and global function with demonstrable prognostic value in multiple settings, including post-myocardial infarction [8] and heart failure [9]. Feature tracking (FT) cardiovascular magnetic resonance imaging (CMR) is a recently developed method to derive strain and wall mechanics, which allows tracking of tissue voxel motion on cine CMR images [10]. Assessment of global longitudinal strain (GLS) and global circumferential strain (GCS) by FT-CMR has been shown to be well-correlated with strain assessed by CMR myocardial tagging [11, 12]. Whilst CMR tagging remains the gold standard for CMR-derived strain, it requires dedicated imaging sequences. Both methods remain generally comparable to speckle-tracking echocardiography but without the field of view limitations [13].

Despite the established prognostic value of CMR-derived strain, there are limited data on its utility for predicting adverse outcomes following ICD implant. Accordingly, the objective of our study was to examine the predictive value of LV strain parameters as measured by FT-CMR on outcomes following primary and secondary prophylactic ICD placement.

## Methods

### Study design

We conducted a retrospective, observational cohort study of patients at two tertiary care hospitals (St. Michael’s Hospital and Sunnybrook Health Sciences Centre, Toronto, Ontario, Canada) between May 2006 and Aug 2017. The study was approved by the Research Ethics Boards at both study sites which waived informed consent.

We included consecutive patients who underwent ICD implantation for primary or secondary prevention of sudden death, were at least 18 years, and had at least one CMR performed prior to device implant. Patients with known diagnoses of arrhythmogenic right ventricular cardiomyopathy and channelopathies (Brugada syndrome, congenital long QT syndrome, catecholaminergic polymorphic ventricular tachycardia) were excluded. Patients with studies that contained incomplete volumetric cine images (n = 32) or insufficient quality for analysis (n = 5) were also excluded. Data on patient demographics, comorbid medical conditions, and medical therapy at time of ICD implant were collected by individual chart review. Appropriate ICD shocks were defined as shock therapies provided to treat a sustained ventricular tachyarrhythmia, including ventricular tachycardia or fibrillation.

### Outcomes

The primary outcome was a composite of appropriate ICD shock or all-cause death. We did not include anti-tachycardia pacing (ATP) as a component of the primary outcome because it is more dependent upon programming and less standardized, therefore less robust than appropriate shock as an endpoint for ICD treatment benefit [14]. We also assessed for the outcome of any appropriate ICD therapy (including ATP) in ancillary analyses.

Patients were followed prospectively in a cardiac implantable electronic device assessment clinic at 6-month intervals or less based on clinical or remote monitoring events. All events were reviewed by trained device technologists and at least one cardiologist, blinded to CMR strain measurements. Deaths were determined using electronic chart review, hospital and autopsy records, or confirmed by the primary care provider. Patients who did not develop the corresponding endpoint by the end of observation period were censored at the last clinic follow-up or medical contact.

### CMR image processing

Baseline CMR imaging was performed on all patients prior to ICD insertion. All CMR examinations were performed with a 1.5T CMR scanner (Intera, Philips Healthcare, Netherlands or TwinSpeed Excite, General Electric Healthcare, Milwaukee, Winsconsin, USA) using a cardiac coil and retrospective electrocardiographic (ECG) gating. The Philips 1.5T scanner used a 5-channel (SENSE) cardiac coil and the GE scanner used an 8-channel cardiac coil.

Standard protocols using validated, commercially available sequences were used. Images were obtained with breath-hold at end-expiration. Standard long-axis two-, four-, and three-chamber views were obtained, as well as sequential short-axis covering the LV. An electrocarddiogram (ECG) gated, breath-hold balanced steady-state free precession (bSSFP) sequence was used to acquire cine images in long-axis planes followed by sequential short-axis cine loops with the following parameters: repetition time 4 ms, time to echo 2 ms, slice thickness 8 mm, field of view 320–330 × 320–330 mm (tailored to achieve optimal spatial resolution and image acquisition time), matrix size 256 × 196, temporal resolution of < 40 ms (depending on the heart rate) and flip angle 50 degrees. Late gadolinium enhancement (LGE) images were obtained for myocardial scar assessment, about 15 min following the administration of gadolinium contrast (Gadobenate Dimeglumine [Multihance; Bracco S.p.A., Milan, Italy] or Gadoteridol [Prohance; Bracco S.p.A.]) at a dose 0.1 mmol/kg, using a 2-D segmented phase-sensitive inversion-recovery gradient echo sequence (repetition time 6.1 ms, time to echo 3.0 ms, flip angle 25 degrees, matrix 512 × 512; inversion time adjusted to null normal myocardium). Prior to image processing, all CMR studies were de-identified and assigned a unique identification code. CMR studies were analyzed with cvi42 (Circle Cardiovascular, Calgary, Alberta, Canada).

LV strain analysis was based on FT-CMR and performed according to previously published methods [15]. The LV endocardial and epicardial borders were manually traced in long-axis and short-axis bSSFP cine images at end-diastole, which were then automatically propagated (tracked) throughout the cardiac cycle. Peak systolic strain is the percent maximum change in length relative to baseline length (Lagrangian strain); a positive strain implies elongation while negative strain implies shortening [16]. Longitudinal strain parameters were obtained from the long-axis orientation (2-chamber, 3-chamber, and 4-chamber views), while circumferential and radial strain parameters were measured from the entire LV stack in short-axis orientation.

Three parameters of LV strain were obtained: global longitudinal strain (GLS), global circumferential strain (GCS), and global radial strain (GRS). Strain expresses the myocardial deformation at end-systole as a percentage of baseline at end-diastole: longitudinal strain measures LV longitudinal shortening from base to apex, circumferential strain measures circumferential shortening of the short-axis of the LV (i.e., more negative GLS and GCS indicate greater shortening, or better systolic function), and radial strain measures myocardial thickening from endocardium to epicardium (i.e., more positive GRS indicates greater thickening, or better systolic function).

LV end-diastolic volumes (LVEDV) and left ventricular end-systolic volumes (LVESV) were measured using the short-axis cine images by manually tracing endocardial contours during ventricular end-diastole and end-systole, using the blood volume method that included papillary muscles and trabeculations. LV mass (LVM) was calculated using the area occupied between the endocardial and epicardial borders multiplied by the sum of the slice thickness and interslice distance, using contiguous short-axis slices at end-diastole, and multiplied by specific gravity of myocardium (1.05). LVEDV, LVESV, and LVM were indexed (LVEDVI, LVESVI, and LVMI, respectively) to body surface area calculated using the Mostellar formula. A single reader measured LV strain in random order, and LV measurements and LGE assessment were performed independently by 2 other CMR readers. All CMR readers were blinded to other clinical characteristics and outcome data.

### Statistical analysis

Continuous variables are presented as means with standard deviation or medians with interquartile range (IQR). The non-parametric Mann–Whitney U test and Kruskal–Wallis test were used to compare continuous data between two and three groups. Chi-square or Fisher’s exact test was used to compare categorical variables between groups. The relationships between CMR parameters were examined using non-parametric Spearman’s correlation test.

Kaplan–Meier curves were constructed for quartiles of LV strain parameters and compared by the log-rank test for trend. Cox proportional hazards models were used to examine the association between CMR LV strain parameters (analyzed as continuous variables) and outcome, adjusting for age, LVEF, ICD indication (primary vs. secondary), and presence or absence (binary variable) LV scar or infarct based on LGE imaging. Variance inflation factors were evaluated for potential collinearity; both GLS and LVEF were permitted to enter into the same multivariable model (variance inflation factors < 2.5). However, because GCS and GRS demonstrated strong correlations with LVEF (variance inflation factors = 3.8 and 3.1, respectively), these variables were not entered into the same models containing LVEF to avoid collinearity. Interaction testing was performed between strain parameters and ICD indication, presence of ischemic heart disease, and LGE. Because strain parameters are recognized to vary by gender [17], we also tested for their interaction. Multivariable regression spline modeling was used to examine potentially non-linearity of covariates. Model discrimination was assessed by Harrell’s c and Somers’ d, and model fit by Akaike’s information criterion. We performed ancillary analyses adjusting for quantitative scar/infarct, and considering the time to first appropriate anti-tachycardia pacing, appropriate ICD shock or death (n = 152) as the outcome.

Intra-observer reproducibility was determined by having the same reader measure a random sample of 20 CMR studies, and intra-class correlation coefficients for absolute agreement were calculated. Statistical significance was defined as a two-sided p value < 0.05. All data were analyzed using SPSS (version 22, Statistical Package for the Social Sciences, International Business Machines, Inc., Armonk, New York, USA) and STATA (version 16.1, Stata Corp, College Station, Texas, USA).

## Results

A total of 364 patients were studied, and their baseline characteristics are presented in Table 1. The mean age of the cohort was 61 years and 79% were male; 61% had an ICD implanted for a primary prevention indication, and 57% had ischemic heart disease. The median time from CMR to ICD implant was 29 days. Over a median follow-up of 62 months (IQR: 39–101), 75 patients (20.6%) had appropriate ICD shocks and 64 patients (17.6%) died; 118 (32.4%) of the cohort reached the primary composite endpoint. Correlations between strain parameters and LVEDVI, LVESVI, LVMI and LVEF are summarized in Table 2. There were negative correlations between LVEF and GLS (lower LVEF correlated with less negative GLS value) and GCS (lower LVEF correlated with less negative GCS value), and positive correlation between LVEF and GRS (lower LVEF correlated with less positive GRS value).

**Table 1:** Baseline characteristics of study patients at time of ICD implant

N	364
Mean age, years (SD)	60.9 (13.5)
Male (%)	78.6
Hypertension (%)	51.4
Diabetes (%)	28.9
Dyslipidemia (%)	48.9
Current smoking (%)	11.8
Prior angina (%)	14.3
Prior myocardial infarction (%)	41.2
Prior stroke (%)	8.0
Prior heart failure (%)	39.6
Prior cardiac arrest (%)	11.6
Prior PCI (%)	21.2
Prior CABG (%)	16.5
Chronic kidney disease (%)	4.9
Primary prevention indication (%)	61.0
Ischemic heart disease (%)	57.4
Dilated cardiomyopathy (%)	25.0
Hypertrophic cardiomyopathy (%)	3.8
Myocarditis (%)	3.3
Sarcoidosis (%)	3.0
Other cardiomyopathy (%)	9.6
Cardiovascular medications (%)	
Aspirin	55.7
Anticoagulant	27.8
ADP receptor inhibitor	15.9
Diuretic	44.0
Beta-blocker	82.3
ACEi	64.4
ARB	11.4
Statin	64.9
Antiarrhythmic	16.2
Amiodarone	12.8
Other	3.4
CMR parameters, mean (SD)	
LVEDVI, mL/m^2^	140.0 (45.4)
LVESVI, mL/m^2^	99.3 (45.4)
LVMI, g/m^2^	70.6 (19.0)
LVEF, %	32.1 (13.8)
GLS, %	− 9.0 (4.3)
GCS, %	− 9.9 (5.0)
GRS, %	19.9 (12.0)

*ACE*i angiotensin converting enzyme inhibitor, *ARB* angiotensin receptor blocker, *CMR* cardiovascular magnetic resonance, *LVEF* left ventricular ejection fraction, *LVESVI* left ventricular end-systolic volume index, *LVEDVI* left ventricular end-diastolic volume index, *LVMI* left ventricular mass index, *GLS* global longitudinal strain, *GCS* global circumferential strain, *GRS* global radial strain, *PCI* percutaneous coronary intervention, *CABG* coronary artery bypass graft surgery, *SD* standard deviation

**Table 2:** Spearman’s correlation coefficients between left ventricular strain parameters and left ventricular volume, mass index, and ejection fraction parameters

	LVEDVI	LVESVI	LVMI	LVEF
GLS	0.48	0.61	0.41	− 0.73
	(p < 0.001)	(p < 0.001)	(p < 0.001)	(p < 0.001)
GCS	0.63	0.75	0.45	− 0.83
	(p < 0.001)	(p < 0.001)	(p < 0.001)	(p < 0.001)
GRS	− 0.56	− 0.69	− 0.41	0.8
	(p < 0.001)	(p < 0.001)	(p < 0.001)	(p < 0.001)

*GLS* global longitudinal strain, *GCS* global circumferential strain, *GRS* global radial strain, *LVEDVI* left ventricular end-diastolic volume index, *LVMI* left ventricular mass index, *LVESVI* left ventricular end-systolic volume index, *LVEF* left ventricular ejection fraction

**Fig. 1:**
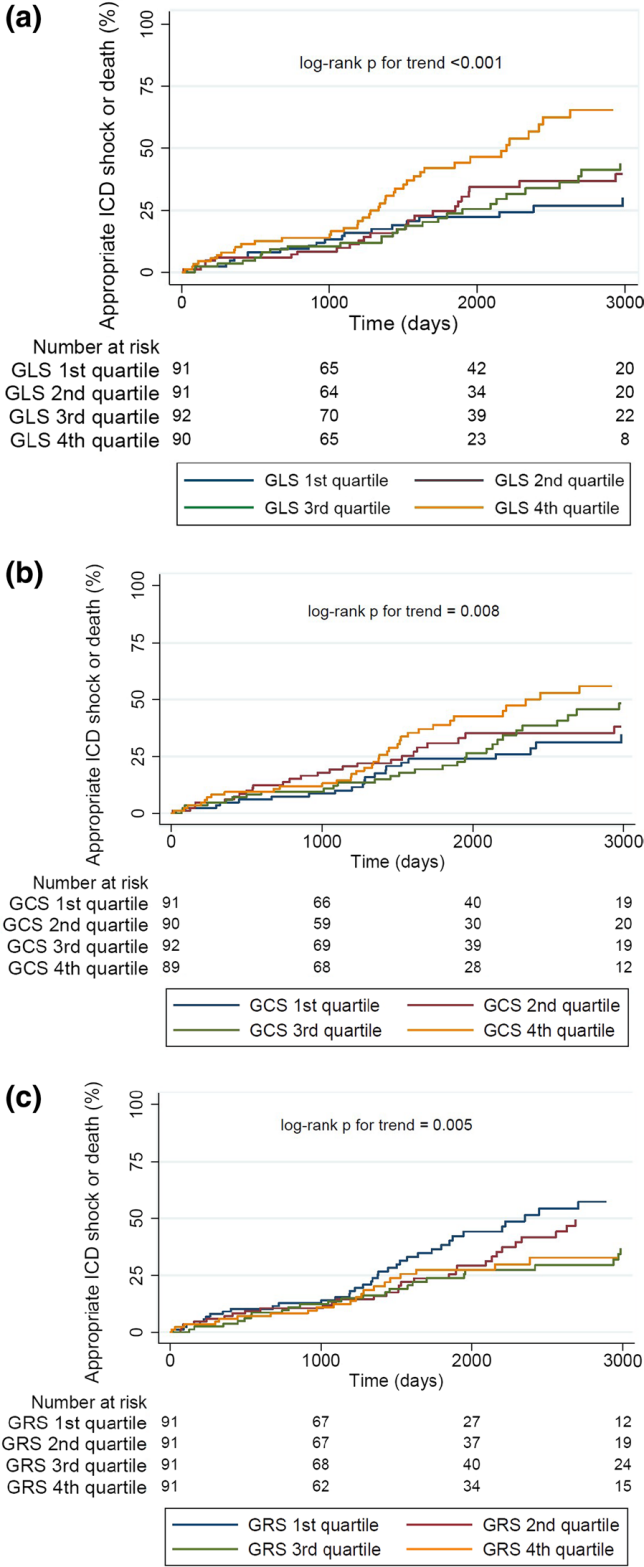
Kaplan–Meier curves for appropriate implantable cardioverter-defibrillator (ICD) shocks or all-cause death stratified by left ventricular (LV) strain cardiovascular magnetic resonance (CMR) parameters divided into quartiles: global longitudinal strain (GLS), global circumferential strain (GCS), and global radial strain (GRS). P for trend reflect differences across all quartiles; 4th quartiles represent the least negative GLS and GCS (less shortening, or worse systolic function) and most positive GRS (greater thickening, or better systolic function)

Figure 1 illustrates the Kaplan–Meier curves of the primary composite endpoint for each strain parameter by quartiles. Significant associations with appropriate ICD shocks or death were demonstrated for GLS (Fig. 1A), GCS (Fig. 1B), and GRS (Fig. 1C) (all p for trend < 0.01). Given the collinearity between strain parameters and LVEF, multivariable analyses excluding LVEF but incorporating age, indication (primary versus secondary prevention), presence of ischemic heart disease, and presence of LGE demonstrated GLS, GCS, and GRS to be independently predictive of both the primary outcome and all-cause mortality (Table 3). When the analyses were stratified by primary versus secondary prevention, the results were similar, and there was no significant interaction (all p for interaction > 0.10). In the multivariable analyses incorporating LVEF, GLS remained a significant independent predictor of the primary endpoint (HR 1.05 per 1% higher GLS, 95% CI 1.01–1.09, p = 0.010) and all-cause mortality (HR 1.06 per 1% higher GLS, 95% CI 1.02–1.11, p = 0.003). With respect to model discrimination, Harrell’s c were 0.600 and 0.613, and Somers’ d were 0.200 and 0.225, respectively, for the models without and with GLS for the primary endpoint; Akaike’s information criterion was lower (i.e., better) for the model including GLS. In a sensitivity analysis adjusting for quantitative LGE by visual assessment, the findings were unchanged (HR 1.05 per 1% higher GLS, 95% CI 1.01–1.09, p = 0.01). GLS was also independently associated with the composite endpoint of appropriate anti-tachycardia pacing, ICD shock or death (HR 1.08 per 1% higher GLS, 95% CI 1.04–1.12, p < 0.001), which was maintained (HR 1.06 per 1% higher GLS, 95% CI 1.01–1.11, p = 0.020) after further adjustment for LVEF.

**Table 3:** Cox proportional hazards regression analysis for the association between clinical and CMR strain parameters and time to first appropriate ICD shock or all-cause mortality, or time to all-cause mortality, stratified by primary and secondary prevention indication*

Strain parameter	Adjusted HR per 1% higher value (95% CI)	p value	Stratified analysis	
			Primary prevention	Secondary prevention
Appropriate ICD shock or all-cause mortality				
GLS	1.07 (1.02–1.11)	0.003	1.14 (1.04–1.24)	1.05 (1.00–1.10)
GCS	1.06 (1.01–1.10)	0.008	1.05 (0.98–1.12)	1.07 (1.01–1.12)
GRS	0.97 (0.95–0.99)	0.012	0.96 (0.93–1.00)	0.98 (0.95–1.00)
All-cause mortality				
GLS	1.08 (1.03–1.13)	0.002	1.15 (1.01–1.31)	1.05 (1.00–1.10)
GCS	1.10 (1.03–1.17)	0.003	1.06 (0.98–1.16)	1.17 (1.08–1.26)
GRS	0.94 (0.90–0.98)	0.004	0.96 (0.89–1.02)	0.92 (0.88–0.97)

*Adjusted for age, primary prevention, ischemic heart disease, presence of late gadolinium enhancement. There was no significant interaction (all p interaction > 0.10) between primary/secondary prevention and any of the LV strain parameters

*GLS* global longitudinal strain, *GCS* global circumferential strain, *GRS* global circumferential strain

Multivariable Cox regression using restricted cubic splines for each strain parameter did not show clear departure from linearity for GLS, GCS, and GRS. There was no significant interaction between GLS and a primary prevention indication for ICD implant (p = 0.32), ischemic heart disease (p = 0.92), presence of LGE (p = 0.86), or gender (p = 0.32).

Intra-observer variability was excellent, with intraclass correlation coefficients for absolute agreement of 0.94 (95% CI 0.85–0.98) for GLS, 0.95 (95% CI 0.83–0.98) for GCS, and 0.95 (95% CI 0.86–0.98) for GRS (all p < 0.001).

## Discussion

In this study of primary and secondary prevention ICD patients with ischemic and non-ischemic cardiomyopathy, CMR-derived LV strain parameters were significantly correlated with LV volumes, LVM, and LVEF. In both univariate and multivariable analyses excluding LVEF for collinearity, reduced GLS, GCS, and GRS values were associated with appropriate ICD shocks and death, as well as all-cause mortality alone. Furthermore, GLS remained a predictor of appropriate ICD shocks and mortality independently of LVEF, regardless of whether the ICD indication was primary or secondary prevention, the presence of ischemic heart disease, or the presence of scar/infarct.

It is recognized that LVEF alone has limited discrimination for predicting sudden death [18]. CMR has an established role in improving risk stratification through improved tissue characterization, specifically focused on both the patterns and quantification of LGE in ischemic and nonischemic cardiomyopathies alike [19, 20]. Although speckle tracking echocardiography derived GLS measurements have demonstrated prognostic value, they are dependent on high quality images. Our study utilized a CMR parameter that does not require additional image acquisition, is rapidly acquired and feasible on almost all routine CMR cine images, and therefore could potentially be used to refine ICD patient selection. As the availability of CMR increases—providing accurate LVEF and right ventricular ejection fraction (RVEF) assessment [21–23], scar quantification, tissue characterization [24], assessment of myocardial viability [25], quantitative valvular assessment and now, LV strain analysis—its role in the noninvasive evaluation of arrhythmia risk will become even more integral.

Myocardial strain is a dimensionless entity, referring to the change in length of myocardium relative to its original length. Subendocardial myocardial fibres are responsible for long-axis contraction, whereas mid-myocardial and sub-epicardial fibres are responsible for circumferential and radial contraction. GLS has demonstrated utility, independent of LVEF, as a sensitive parameter of LV function predictive of clinical outcomes across a wide spectrum of cardiac diseases, including myocardial infarction, heart failure, detection and monitoring of chemotherapy cardiotoxicity, and valvular heart disease [26, 27]. In regards to its ability to predict adverse arrhythmic outcomes, it is conceivable that anatomic abnormalities that predispose to arrhythmia (for example, due to heterogeneity in conduction through myocardial scar) also result in heterogeneous regional contraction, which in turn is reflected in strain parameters. LV strain is a less load-dependent measure of contractile function than LVEF and a more sensitive measure of impaired contractility. Furthermore, derangements in regional contractility, induced by diffuse, as opposed to focal myocardial fibrosis, may be identified using FT-CMR derived strain. Indeed, echocardiography-derived mechanical dispersion (a parameter determined from myocardial strain measurements) has been demonstrated to have incremental predictive value, independent of LVEF, for ventricular arrhythmias [28].

Methods of assessing strain by CMR include tissue phase mapping, strain-encoded imaging, myocardial tissue tagging, displacement encoding with simulated echoes, and FT-bSSFP imaging [29]. Although advances in myocardial tissue tagging with methods such as harmonic phase imaging and strain-encoded imaging have significantly reduced analysis time, acquisition and post-processing of additional sequences are still required. In contrast, FT-CMR uses semi-automated volumetric measures on routinely acquired cine bSSFP images. It is therefore available across different CMR vendors and field strengths, and does not require any image conversion to alternative formats or dedicated pulse sequences. Although segmental strain has been shown to be heterogeneous with FT-CMR [10, 30], global strain parameters appear to be more robust and reproducible.

Our findings add to existing evidence supporting the prognostic significance of CMR-derived strain measurements. Romano et al. examined 1012 patients with LV dysfunction (LVEF < 50%) using FT-CMR derived GLS for predicting all-cause mortality, and determined that it afforded incremental prognostication for all-cause mortality beyond LVEF and LGE in both ischemic and non-ischemic cardiomyopathy subgroups [31]. Mordi et al. found GCS derived by harmonic phase myocardial tagging to be independently prognostic in their single-centre study of 539 unselected patients undergoing CMR after adjusting for LVEF and LGE [32]. Paiman et al. found the extent of moderately impaired LV GCS to be associated with appropriate ICD therapy in 121 patients with primary prevention ICDs and history of previous myocardial infarction [33]. However, this original study was relatively small with only 30 events and, the endpoint of appropriate ICD therapy included ATP in addition to shocks. Furthermore, other global strain parameters were not assessed, and patients with non-ischemic cardiomyopathy were not included. Our study extends the prognostic value of LV strain to ICD recipients, and may have a future role in directing primary prevention ICD treatment to patients more likely to derive benefit than those selected based only on current risk stratification models which are predominantly influenced by LVEF.

We selected a primary composite endpoint encompassing arrhythmia (appropriate ICD shock) and all-cause mortality with the aim of determining whether strain could help refine ICD treatment benefit. It must be acknowledged that the threshold for an ICD shock is, to some degree, dependent upon device programming, and that not all ICD shocks, even when deemed appropriate, are life-saving [7]. However, adjudicated appropriate shocks remain the most robust endpoint besides death for determining that patients derived survival benefit from the ICD. While patients with ICDs are also at elevated risk of non-arrhythmic death [34], the cause of death can be particularly difficult to ascertain in both randomized and observational studies, despite central adjudication. Our study is consistent with randomized trials of ICDs that have used all-cause, rather than disease-specific, mortality.

We had a relatively long follow-up period in this study with > 100 events, and our cohort had relatively few exclusion criteria, enhancing the generalizability of our results. All CMR measurements were performed independently by readers blinded to clinical outcomes. We focused on appropriate ICD shocks that were independently adjudicated, a more robust endpoint for survival than including either any shock or anti-tachycardia pacing [35, 36]. Similarly, we selected all-cause mortality over disease-specific mortality, consistent with the landmark trials evaluating ICDs for primary or secondary prevention, as the more clinically relevant endpoint.

### Limitations

Several limitations of the study are acknowledged. Data were not available on New York Heart Association classification of functional status or other clinical parameters, which might be relevant confounders. We only examined a population of patients who were deemed by their treating physician to be at an increased arrhythmia risk to warrant an ICD implant, and the utility of CMR-derived LV strain parameters for risk prediction of malignant arrhythmias in a broader population is unknown. We did not have data from concurrent echocardiographic studies for direct comparison. Selection bias might have been present with patients who were unable to undergo an CMR examination due to severe claustrophobia, renal disease, large body size, pre-existing implantable devices, or who were not deemed by their treating physicians to require CMR as a component of care. The relatively low model discrimination suggests that prediction of appropriate ICD treatment remains challenging, and more refined risk stratification tools are needed. Finally, the relative prognostic significance of LV strain and tissue mapping could not be determined in this study. Our study provides impetus for further investigation into the role of GLS as a novel arrhythmia risk marker. Randomized studies examining the incremental value of strain beyond LVEF may be particularly informative in those with borderline LVEF (35–40%) being considered for primary prevention ICD, or non-ischemic cardiomyopathy with LVEF < 35% in view of recent trial evidence calling to question the benefit of routine ICD implant in this population[37].

## Conclusions

Among patients who underwent ICD implant for primary and secondary prevention of sudden cardiac death, FT-CMR GLS assessment was independently predictive of appropriate shocks and all-cause mortality, even after adjustment for LVEF. These findings support the potential use of FT-CMR derived strain analyses to enhance the selection of patients most likely to benefit from ICD implant. Further studies are needed to confirm these findings in a broader spectrum of ICD candidates.

## Data Availability

The datasets generated and/or analyzed during the current study are not publicly available due to requirement for data sharing agreement but are available from the corresponding author on reasonable request.

## Abbreviations

ATP: Anti-tachycardia pacing
bSSFP: Balanced steady state free precession
CMR: Cardiovascular magnetic resonance
ECG: Electrocardiogram
FT: Feature tracking
GCS: Global circumferential strain
GLS: Global longitudinal strain
GRS: Global radial strain
ICD: Implantable cardioverter-defibrillator
LGE: Late gadolinium enhancement
LV: Left ventricle/left ventricular
LVEDV: Left ventricular end-diastolic volume
LVEDVI: Left ventricular end-diastolic volume index
LVEF: Left ventricular ejection fraction
LVESV: Left ventricular end-systolic volume
LVESVI: Left ventricular end-systolic volume index
LVM: Left ventricular mass
LVMI: Left ventricular mass index
RVEF: Right ventricular ejection fraction
